# Statins in Depression: An Evidence-Based Overview of Mechanisms and Clinical Studies

**DOI:** 10.3389/fpsyt.2021.702617

**Published:** 2021-07-27

**Authors:** Riccardo De Giorgi, Nicola Rizzo Pesci, Alice Quinton, Franco De Crescenzo, Philip J. Cowen, Catherine J. Harmer

**Affiliations:** ^1^Department of Psychiatry, Warneford Hospital, University of Oxford, Oxford, United Kingdom; ^2^Oxford Health NHS Foundation Trust, Warneford Hospital, Oxford, United Kingdom; ^3^Department of Neurosciences “Rita Levi Montalcini,” San Luigi Gonzaga University Hospital, University of Turin, Turin, Italy

**Keywords:** statin, HMG 3-hydroxy-3-methylglutaryl, depression, antidepressant, mechanism, review

## Abstract

**Background:** Depression is a leading cause of disability, burdened by high levels of non-response to conventional antidepressants. Novel therapeutic strategies targeting non-monoaminergic pathways are sorely needed. The widely available and safe statins have several putative mechanisms of action, especially anti-inflammatory, which make them ideal candidates for repurposing in the treatment of depression. A large number of articles has been published on this topic. The aim of this study is to assess this literature according to evidence-based medicine principles to inform clinical practise and research.

**Methods:** We performed a systematic review of the electronic databases MEDLINE, CENTRAL, Web of Science, CINAHL, and ClinicalTrials.gov, and an unstructured Google Scholar and manual search, until the 9th of April 2021, for all types of clinical studies assessing the effects of statins in depression.

**Results:** Seventy-two studies were retrieved that investigated the effects of statins on the risk of developing depression or on depressive symptoms in both depressed and non-depressed populations. Fifteen studies specifically addressed the effects of statins on inflammatory-related symptoms of anhedonia, psychomotor retardation, anxiety, and sleep disturbances in depression. Most studies suggested a positive effect of statins on the occurrence and severity of depression, with fewer studies showing no effect, while a minority indicated some negative effects.

**Limitations:** We provide a narrative report on all the included studies but did not perform any quantitative analysis, which limits the strength of our conclusions.

**Conclusions:** Robust evidence indicates that statins are unlikely to lead to depressive symptoms in the general population. Promising data suggest a potential role for statins in the treatment of depression. Further clinical studies are needed, especially in specific subgroups of patients identified by pre-treatment assessments of inflammatory and lipid profiles.

## Introduction

### 1a Depression

Depression is a major contributor to the worldwide burden of disease (1). First-line antidepressant treatments are widely accessible but hampered by some critical issues: significant side effects, delayed therapeutic onset, and limited efficacy (2). Indeed the response rate of antidepressants ranges between 50 and 60% (3), and about one-third of depressed patients remain symptomatic after four treatment steps over one year (4). Most of the currently used antidepressants primarily affect monoaminergic (i.e., serotonin or 5-hydroxytryptamine, 5HT; noradrenaline, NA; dopamine, DA) neurotransmission (5), and scarce progress has been made over the last several years to develop novel antidepressant drugs. An innovative and promising approach favours instead the repurposing of existing medication with a well-defined safety profile and capable of targeting emerging physiopathological pathways implicated in depression (6).

Systemic and central nervous system (CNS) inflammatory processes appear causally involved in at least certain subtypes of depressive disorders (7). Inflammatory molecules such as C-reactive protein (CRP) and interleukin (IL)-6 are increased in depressed patients' peripheral blood (8, 9) and cerebrospinal fluid. Moreover, the prevalence of depression is higher among patients suffering from immune-mediated inflammatory disorders (10), and immunomodulation improves their depressive symptomatology irrespective of their effects on physical illness (11). Increased inflammatory markers have also been associated with specific subgroups of depressed patients, particularly those responding poorly to conventional antidepressants (12, 13), and those with high levels of anxiety (14), sleep disturbance (15), anhedonia (16), and psychomotor retardation (17, 18) — a cluster of symptoms that have been referred to as “depressive-inflammatory.” Therefore, targeting inflammation in depression may be a viable treatment strategy, and recent meta-analyses have described encouraging effects of anti-inflammatory agents as adjunctive treatments in depressed patients (19).

### 1b Statins in Depression

**Figure 1:**
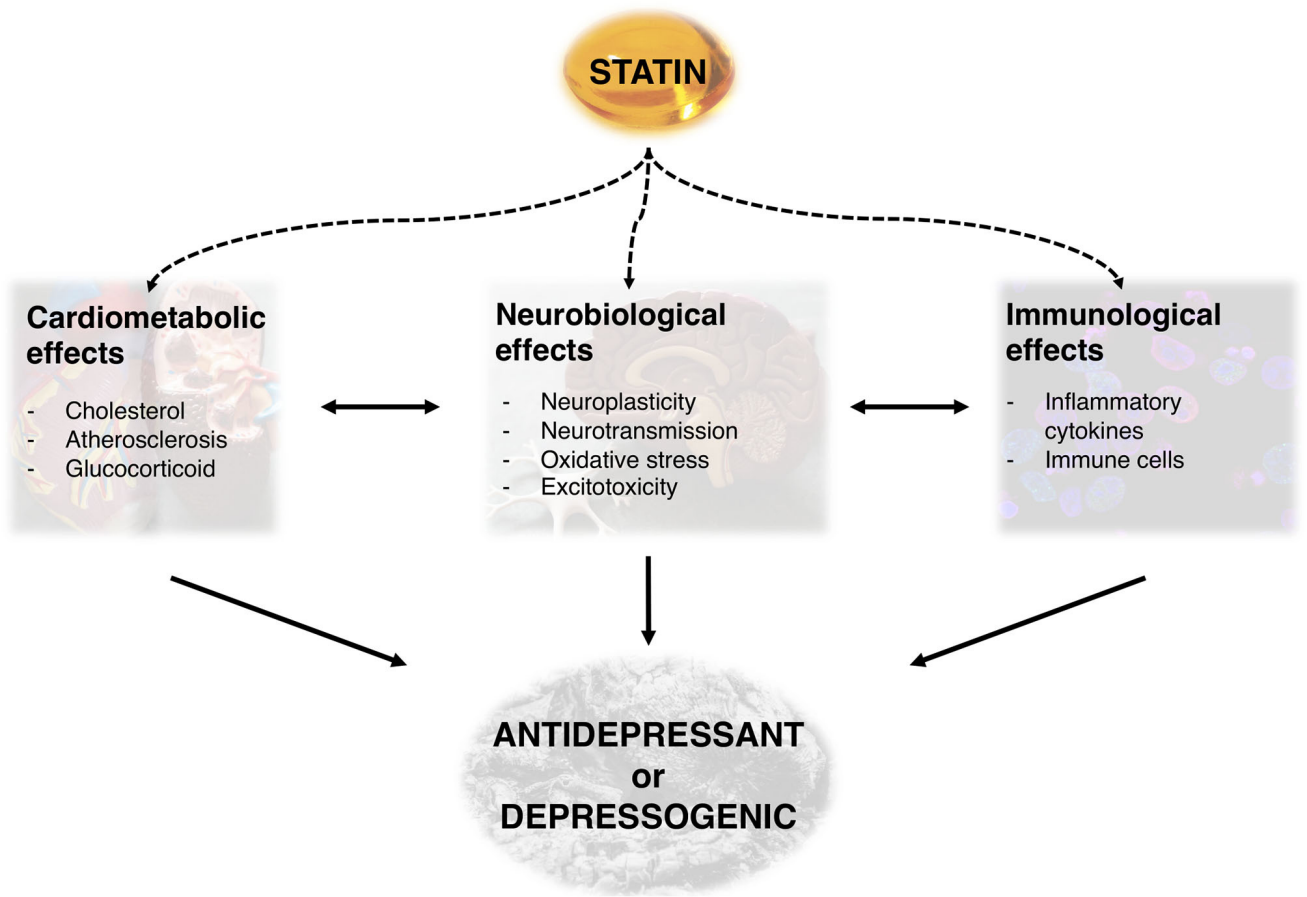
Overview of potential mechanisms underlying the antidepressant or depressogenic effects of statins.

Statins or 3-hydroxy-3-methylglutaryl-Coenzyme A (HMG-CoA) reductase inhibitors are a class of medications capable of reducing blood cholesterol and a mainstay treatment for several cardiovascular, cerebrovascular, and metabolic disorders (20). Thanks to their well-established safety profile (21) and their ability to modulate inflammatory processes (22), they are considered excellent candidates for repurposing in the treatment of depression (23). Indeed, statins seem to possess numerous, and sometimes clashing neurobiological, cardiometabolic, and immunological effects (Figure 1) that might explain both their antidepressant and depressogenic properties (24), as we describe in the paragraphs below.

Firstly, the primary effect of statins on lipid metabolism, extensively interconnected with inflammatory processes (25), may have profound influences on the anatomy and physiology of the nervous system (26), and potentially interact with neurobiological pathways implicated in depression (27) and antidepressant mechanisms (28, 29). Moreover, *in vitro* studies show that statin-mediated cholesterol depletion alters 5HT_1A_ receptor dynamics (30) and impairs the formation and release of synaptic vesicles (31). Some authors also suggest that, on the basis of the “vascular depression hypothesis” (32), the antidepressant effects of statins can be explained by their potential to prevent cerebrovascular accidents and therefore improve quality of life (33).

Statins however can affect neuronal homeostasis in several other ways that are not directly due to their action on cholesterol. There is extensive evidence that inflammation (34) and hypothalamic-pituitary-adrenal (HPA) (35) axis disturbances play a role in the pathophysiology of depression. The anti-inflammatory effects of statins are rapid (36) and independent of their lipid-lowering properties (37). Animal studies show that statins reduce depressive-like symptoms by reducing hippocampal neuroinflammation (38) and more broadly by inhibiting cytokine release in the central nervous system (CNS) and countering microglial and astrocyte activation (39–41). Likewise, human studies suggest that statins might affect mood by offsetting the peripheral pro-inflammatory effects of IL-6 and IL-18 (42). Statins also seem to reduce glucocorticoid levels in rats (43), although they increase serum cortisol in humans (44), which could be associated to the onset depressive symptoms. The depressive and anxiety behaviours caused in rats by chronic mild stress (45) or high-fat diet (46) were counteracted by statins, similarly to antidepressant medications. The depressogenic effect of reactive oxygen species (ROS) in the brain (47) could be reduced by statins both directly (48) and via peroxisome proliferator-activated receptor (PPAR)-γ activity and decreased nitrous oxide (NO) levels (49, 50) according to animal studies. Furthermore, glutamatergic N-methyl-D-aspartate (NMDA)-induced neuronal damage (51), whose modulation is associated to the mechanism of action of ketamine (52), is similarly affected by statins via direct (53) and PI3K/AKT/GSK3b/mTOR-mediated (54) antagonism.

Aside from these broadly neuroprotective effects, statins may also promote hippocampal neuroplasticity, especially implicated in the pathophysiology of depression and response to antidepressant treatment (55), through the increase of brain-derived neurotrophic factor (BDNF) via direct (53, 56) and tissue plasminogen activator (tPA) (57–59) and agmatine/imidazoline (60) pathways. However, higher hippocampal BDNF has also been associated to increased anxious behaviour in rats treated with statins (61).

Finally, the effects of statins on neurotransmitter turnover have also been reviewed. Conventional antidepressants act predominantly by modulating monoamines (dopamine, noradrenaline, serotonin) in the synaptic cleft (62). In this context, statins increase hippocampal serotonin levels (43), and induce serotonergic-dependent antidepressant-like effect that are counteracted by 5HT_1A_ and 5HT2_A/C_ receptor antagonists (63) and upregulate pre-frontal dopamine receptors expression (64) in animal models of depression. Moreover, statins seem to directly potentiate the serotonergic effects of some antidepressants in animals (65–67) and possibly in human trials (68). Non-monoaminergic pathways have also been explored in animal models of depression, showing that statins may elicit antidepressant action via opioid- (69) and endocannabinoid-mediated (70) neurotransmission.

Most of the evidence presented above comes from *in vitro* or animal studies, probably since many of the proposed antidepressant mechanisms of statins, especially in the CNS, are difficult to investigate in humans (24). Human clinical studies have been less consistent and reported both antidepressant and depressogenic effects.

### 1c Aim of the Study

Our review aims to systematically research and describe the literature regarding the role of statins in depression both in the general population and in depressed patients. Therefore, we searched for any studies that investigated the use of or exposure to statins in both depressed and non-depressed participants and their association with the risk of developing depression or their effect on depressive symptoms scores. We also specifically retrieved studies exploring the effect of statins on the symptom domains that appear associated with an inflammatory phenotype of depression, namely anhedonia, psychomotor retardation, anxiety, and sleep disturbance. This enabled us to discuss the available data and propose ways that it could be complemented by research.

## Methods

We conducted an extensive literature search of the PubMed/MEDLINE, Cochrane CENTRAL, ISI Web of Science, CINAHL, and ClinicalTrials.gov databases from the date of inception until the 9th of April 2021, including non-English language articles. We used a well-validated search algorithm (PROSPERO international prospective register of systematic reviews reference CRD42020170938, available at https://www.crd.york.ac.uk/PROSPERO/display_record.php?RecordID=170938) based on our previously conducted systematic review and meta-analysis of randomised controlled trials (71), which combined all the relevant terms for statins, depression, and antidepressants (Supplementary Material). References of the included papers were manually screened for further relevant material, and an unstructured search of Google Scholar was performed for additional grey literature. We contacted the corresponding authors to obtain information about unpublished or incomplete trials as reported on ClinicalTrials.gov.

All studies that reported on any statin and their effect on depression, mood scores, or depressive-inflammatory symptoms in depressed patients were included. We excluded *in vitro* and animal studies, though we summarised these in the introduction. We did not exclude on the basis of study design, comorbidity, concurrent medication use, outcome measures, or length of follow-up as not to compromise the inclusiveness of the review. We excluded previous narrative reviews, commentaries, protocols, and articles that did not report on the intervention/exposure (i.e., any statin) or outcome (i.e., depression, mood scores, depressive-inflammatory symptoms in depressed patients) of interest.

Three researchers (AQ, NRP, RDG) independently screened titles and abstracts for relevance and assessed the full texts for eligibility. Disagreements were discussed with a fourth researcher (FDC) and resolved by consensus. For the included studies, two researchers (AQ, NRP) extracted data about authors' names, year of publication, study design, sample size and characteristics, intervention/exposure, comparison, length of follow-up, primary outcome measures, and point estimates.

Finally, we identified four areas of study (i.e., the effects of statins on the risk of developing depression in non-depressed patients; on depressive symptoms scores in non-depressed patients; on the risk of developing depressive episodes or depressive symptoms scores in depressed patients; on depressive-inflammatory symptoms in depressed patients), and narratively described each article in its context.

## Results

### 3a Studies Included

**Figure 2:**
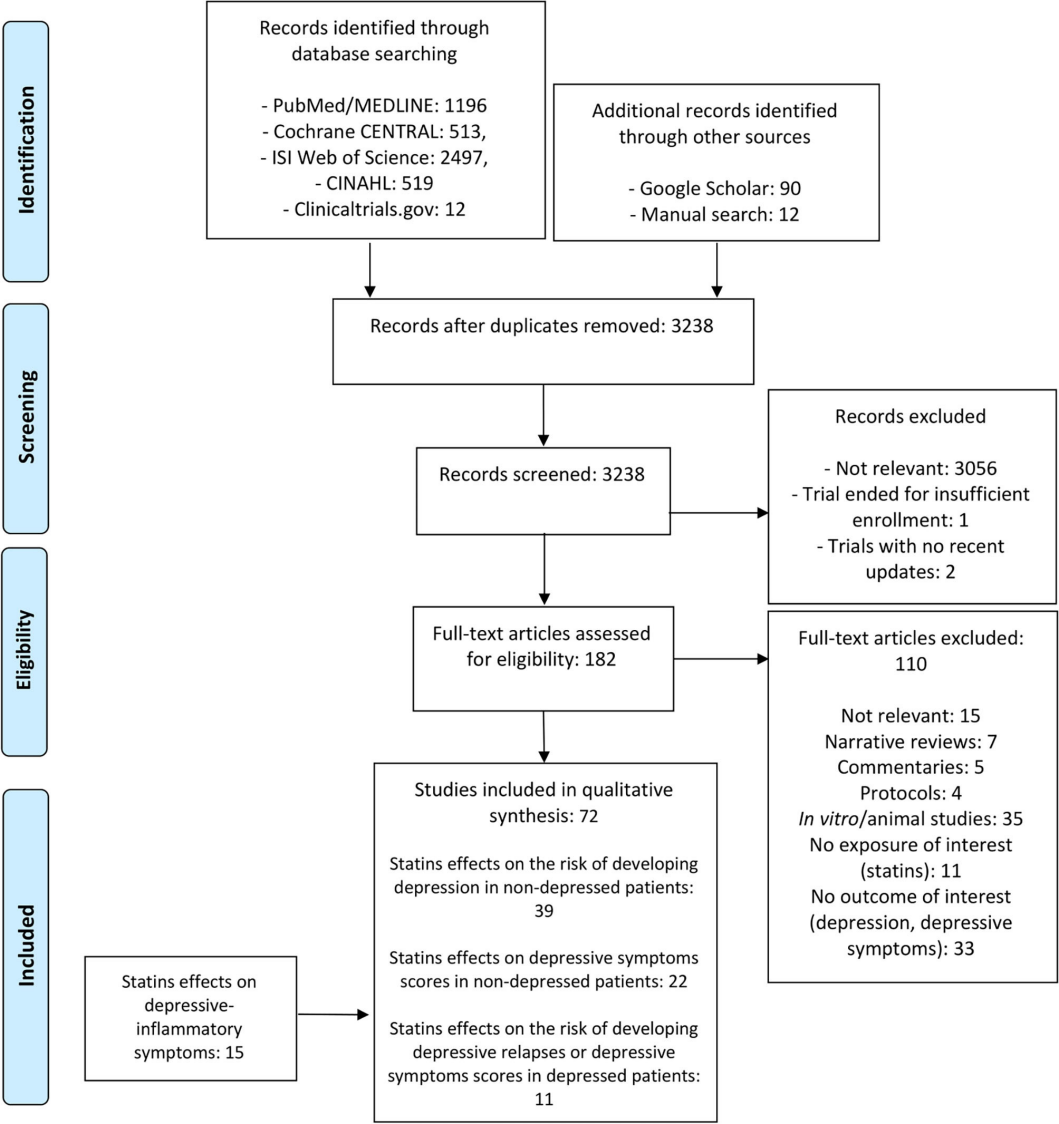
Flow chart of the included/excluded studies.

Our search was reported according to the Preferred Reporting Items for Systematic Reviews and Meta-Analyses (PRISMA) guideline (Figure 2) and retrieved 4,725 records from electronic databases (PubMed/MEDLINE = 1,196, Cochrane CENTRAL = 513, ISI Web of Science = 2,497, CINAHL = 519, Clinicaltrials.gov = 12) and further 102 papers from the Google Scholar and manual searches. After duplicates removal (= 1,589), 3,238 titles and abstracts were screened, of which 3,056 were excluded due to lack of relevance. The remaining 182 articles were assessed in full; a total of 110 were excluded as narrative reviews without meta-analysis (=7), commentaries (=5), protocols (=4), non-relevant (=15), *in vitro*/animal studies (=35), or lacking the intervention/exposure (= 11) or outcome (=33) of interest. The remaining 72 studies (Tables 1–**3**) were divided into three categories: effects of statins on the risk of developing depression in non-depressed patients (=39); effects of statins on depressive symptoms scores in non-depressed patients (=22); effects of statins on the risk of developing depressive episodes or depressive symptoms scores in depressed patients (=11); and a fourth category (**Table 4**) which included 15 studies that specifically investigated effects of statins on depressive-inflammatory symptoms in depressed patients. For all the included studies, we first attempted to report the effect sizes, either extracted from the study or calculated by the authors. Where this was not possible, we report the raw data available in the individual study.

**Table 1:** Overview of studies regarding the effects of statins on depression diagnosis in non-depressed participants.

**Publication**	**Study design**	**Population**	**Exposure**	**Comparison**	**Follow-up**	**Primary outcomes**	Major Findings^*^	**Association**
**Meta-analysis**								
Lee et al. (72)	Meta-analysis	13 observational studies, 5,035,070 participants	Statin use	No use	NR	Diagnosis of depression	OR = 0.87 95% CI = 0.74 to 1.02 *P* = NR	=
Parsaik et al. (73)	Meta-analysis	7 observational studies, 9,187 participants	Statin use	No use	5 years	Diagnosis of depression	OR = 0.68 95% CI = 0.52–0.89 *P* = 0.01	+
**Cohort studies**								
Asplund and Eriksson (74)	Cohort	70,706? ischemic stroke patients	Discharge on statin	No prescription	3 months	Diagnosis of post-stroke depression or antidepressant prescription (self-reported)	OR = 0.99 95% CI = 0.95–1.03 *P* = NR	=
Chuang et al. (75)	Historical cohort	26,852 hyperlipidaemic patients	Statin use	No use	4 years	Diagnosis of depression	HR = 0.81 95% CI = 0.69–0.96 *P* < 0.05	+
Dave et al. (76)	Historical cohort	299,298 statin users	Lipophilic statin use (atorvastatin, lovastatin, simvastatin)	Hydrophilic statin use (pravastatin, rosuvastatin)	3 years	Diagnosis of depression	Lipophilic vs. hydrophilic statins: HR = 1.05 95% CI = 1.00–1.10 *P* = 0.078 Simvastatin vs. hydrophilic statins: HR = 1.09 95% CI = 1.02–1.16 *P* = 0.003	= / -
Glaus et al. (77)	Cohort	1,631 adults aged 35–66 years	Statin use (self-reported)	No use	5.2 years	Diagnosis of depression	HR = 1.25 95% CI = 0.73–2.14 *P* > 0.05	=
Huang et al. (78)	Cohort	408 HNC hyperlipidaemic patients	Statin use	No use	1 year	Diagnosis of depression	HR = 0.85 95% CI = 0.46–1.57 *P* = 0.4252	=
Kang et al (79)	Cohort	286 ischemic stroke patients	Statin use	No use	1 year	Diagnosis of post-stroke depression	Wald = 8.477 95% CI = NR *P* = 0.004	+
Kang et al. (80)	Cohort	11,218 ischemic stroke patients	Statin use	No use	1 year	Diagnosis of post-stroke depression	HR = 1.59 95% CI = 1.30–1.95 *P* < 0.001	-
Kessing et al. (81)	Historical cohort	497,080 statin users	Statin use	No use	Up to 21 years	Diagnosis of depression or antidepressant prescription	Trend test for statin prescription = 0.92 95% CI = 0.92–0.95 *P* < 0.001	+
Kim et al. (42)	Cohort	711 ACS patients	Statin use	No use	1 year	Diagnosis of depression	Prevalence difference = −9.5% (statin users) 95% CI = NR *P* = 0.037	+
Kim et al. (82)	Cohort	288 ischemic stroke patients	Statin use	No use	1 year	Diagnosis of post-stroke depression	OR = 0.54 95% CI = 0.49–0.87 *P* = NR	+
Khokhar et al. (83)	Historical cohort	100,515 TBI patients aged >/=65 years	Statin use	No use	6 months	Diagnosis of depression	RR = 0.85 95% CI = 0.79–0.90 *P* = NR	+
Köhler-Forsberg et al. (84)	Historical cohort	387,954 adults	Statin prescription redemption	No prescription	6.8 years	Diagnosis of depression	HR = 1.33 95% CI = 1.31–1.35 *P* = NR	-
Mansi et al. (85)	Historical cohort	13,944 adults aged 30–85 years	Statin use	No use	4.5 years	Diagnosis of mood disorder	OR = 1.02 95% CI = 0.94–1.11 *P* = 0.6	=
Medici et al. (86)	Cohort	12,176 ICU patients	Statin use	No use	3 years	Diagnosis of depression or antidepressant prescription	RR = 1.04 95% CI = 0.96–1.13 *P* = NR	=
Molero et al. (87)	Historical cohort	1,149,384 statin-users aged >/=15 years	Statin use	No use	8 years	Diagnosis of depressive episode (unplanned hospital visit or specialised outpatient care)	HR = 0.91 95% CI = 0.88–0.94 *P* = NR	+
Otte et al. (88)	Cohort	776 CAD patients	Statin use	No use	6 years	Diagnosis of depression (PHQ>/=10)	OR = 0.62 95% CI = 0.41–0.95 *P* = 0.026	+
	Cross sectional	965 CAD patients	Statin use	No use	–	Diagnosis of depression (PHQ>/=10)	OR = 0.66 95% CI = 0.45–0.98 *P* = 0.04	+
Pasco et al. (89)	Historical cohort	345 women aged >/=50 years	Statin or aspirin use (self-reported)	No use	10 years	Diagnosis of depression (first episode)	HR = 0.20 95% CI = 0.04–0.85 *P* = 0.03	+
	Case-control	345 women aged >/=50 years	Statin use prior to depression onset (self-reported)	No use	10 years	Diagnosis of depression (first episode)	OR = 0.13 95% CI = 0.02–1.02 *P* > 0.05	=
Redlich et al. (90)	Cohort	4,607,990 adults aged >/=40 years	Statin prescription	No prescription	3 years	Diagnosis of depression	OR = 0.92 95% CI = 0.89–0.96 *P* = 0.016	+
Smeeth et al. (91)	Cohort	729,529 adults aged >/=40 years	Statin prescription	No prescription	4.3 years	New antidepressant prescription	HR = 1.01 99% CI = 0.96–1.06 *P* = NR	=
Stafford and Berk (92)	Cohort	193 MI, PTCA, or CABG hospitalised patients	Statin prescription	No prescription	9 months	Diagnosis of depression	OR = 0.21 95% CI = 0.052–0.876 *P* = 0.032	**+**
Wee et al. (93)	Cohort	3,792 TBI patients	Statin use in hyperlipidaemia (SHL)	Untreated hyperlipidaemia (UHL) or normolipidaemia (NL)	3 years	Diagnosis of depression	SHL vs. NL HR= 1.02 95% CI = 0.55–1.89 *P* = 0.9611 SHL vs. UHL HR = 0.63 95% CI = 0.34–1.17 *P* = 0.1433	=
Williams et al. (94)	Historical cohort	836 adult men	Statin or aspirin use	No use	6 years	Diagnosis of mood disorder	HR = 0.55 95% CI = 0.23–1.32 *P* = 0.18	=
	Case-control	937 adult men	Statin or aspirin use	No use	–	Diagnosis of mood disorder	OR = 0.1 95% CI = 0.1–0.4 *P* < 0.001	+
Wium-Andersen et al. (95)	Historical cohort	91,842 ACS patients, 91,860 matched individuals	Statin use	No use	12 years	Diagnosis of early (<1 year) or late (1–12 years) depression or antidepressant prescription	Early depression ACS patients: HR = 0.94 95% CI = 0.86–0.94 *P* = NR Non ACS: HR = 1.04 95% CI = 0.96–1.12 *P* = NR Late depression ACS patients: HR = 0.96 95% CI = 0.82–0.90 *P* = NR Non ACS: HR = 1.00 95% CI = 0.95–1.06 *P* = NR	**+**
Wium-Andersen et al. (96)	Historical cohort	147,487 ischemic stroke patients, 160,235 matched individuals	Statin use	No use	1 year	Diagnosis of early (<1 year) or late (1–12 years) depression or antidepressant prescription	Early depression stroke patients: HR = 0.71 95% CI = 0.70–0.73 *P* = NR Non stroke: HR = 1.00 95% CI = 0.94–1.05 *P* = NR Late depression stroke patients: HR = 0.90 95% CI = 0.87–0.93 *P* = NR Non stroke: HR = 0.90 95% CI = 0.86–0.94 *P* = NR	+
Yeh et al. (97)	Historical cohort	9,139 Asthma-COPD overlap syndrome patients	Statin use	No use	Up to 11 years	Diagnosis of depression	HR = 0.36 95% CI = 0.25–0.53 *P* < 0.001	+
Young-Xu et al. (98)	Cohort	371 CAD patients	Statin use	No use	4 years	Diagnosis of depression (Kellner Symptom questionnaire >/=7)	OR = 0.63 95% CI = 0.43–0.93 *P* = NR	+
**Case-control studies**								
Yang (33)	Case-control	366 hyperlipidaemic patients aged 40–79 years	Statin use	No use	–	Diagnosis of depression	OR = 0.4 95% CI = 0.2–0.9 *P* < 0.05	+
**Cross-sectional studies**								
Agustini et al. (99)	Cross sectional	19,114 community-dwelling participants aged >/= 70 years	Statin use (self-reported)	No use	–	Diagnosis of depression (CES-D >/= 8)	OR = 1.09 95% CI = 0.98–1.20 *P* = 0.11	**=**
Boumendil and Tubert-Bitter (100)	Cross sectional	17,244 adults	Simvastatin use (self-reported)	No use	–	Absenteeism due to depression	PR = 2.18 95% CI = 1.18–4.03 *P* = NR	–
Feng et al. (101)	Cross sectional	2,804 adults aged >/= 55 years	Statin use (self-reported)	No use	–	Diagnosis of depression (GDS >/=5)	OR = 0.71 95% CI = 0.52–0.97 *P* = NR	+
Lindberg and Hallas (102)	Cross sectional	166 users of antidepressant and statins	Statin use	No prescription	–	Antidepressant prescription redemption before vs after redemption of statin	RR= 1.06 95% CI = 0.79 to 1.45 P= NR	=
Williams et al. (103)	Cross sectional	638 White Europeans, 695 South Asians and African-Caribbean	Statin prescription	No prescription	–	Diagnosis of depression (GDS >/=4)	White Europeans: OR = 0.54 95% CI = 0.26 to 1.13 *P* = NR South Asian and African-Caribbean: OR = 1.67 95% CI = 0.97–2.88 *P* = NR	=
**Case series**								
Cham et al. (104)	Case series	11 male and 1 female patients	Statin treatment (simvastatin, atorvastatin, rosuvastatin, lovastatin, pravastatin) for 1 day to several months	–	–	–	Episodes of violent ideation, irritability, depression and suicide were reported. All 12 cases resolved upon discontinuation and recurred with re-challenge when attempted. Four cases met Naranjo criteria for definite causality, 4 for probable causality, 4 for possible causality.	–
Duits and Bos (105)	Case series	4 female patients aged 32–59	Simvastatin	–	–	–	Two patients developed psychotic, obsessive, depressive symptoms and suicidal/homicidal thoughts, and required treatment with clomipramine and cognitive therapy. One patient developed paranoid thoughts, suicidality, agitation and depressive symptoms after 4 days of simvastatin; symptoms resolved upon discontinuation. One patient suffered a depressive syndrome with psychotic features after 3 months of simvastatin; management with discontinuation, antipsychotic and antihypertensive medications was necessary.	**–**
Lechleitner et al. (106)	Case series	4 female patients with primary hypercholesterolaemia, aged 44–66	Pravastatin 10 mg for 12 weeks	-	-	-	Three patients developed mild-moderate depressive symptoms reversed by discontinuation. One patient developed severe psychiatric symptoms and suicidality, improved on discontinuation and didn't reoccurred with lovastatin treatment.	**-**
Rosenson Goranson (107)	Case series	2 male hyperlipidaemic patients, aged 51–53	Lovastatin 20–60 mg/die	–	–	–	Two patients developed sleep disturbances, anxious mood and irritability after several weeks of lovastatin treatment (20 mg/die and 60 mg/die); symptoms reversed 48 h after discontinuation, and reoccurred upon re-challenge, but not upon starting of pravastatin treatment.	**–**
Tatley and Savage (108)	Case series	Adverse reaction reports to New Zealand Centre for Adverse Reaction Monitoring	Statin treatment (simvastatin, atorvastatin, Fluvastatin, pravastatin)	–	–	–	203 reports of psychiatric adverse events associated with statins (67 reports of mood disorders, 30 of cognitive disorders, 51 of sleep disorders, 14 of perception disorders, 107 other reactions such as asthenia, fatigue, lethargy). 57 reactions were severe. 34 had documented recurrence upon re-challenge	**–**

* *The effect size of the main findings, either extracted from the study or calculated by the authors. Where this was not possible, we report the raw data*.

*ACS, acute coronary syndrome; CABG, coronary artery bypass graft; CAD, coronary artery disease; CES-D, centre for epidemiological studies – depression scale; CI, confidence interval; COPD, chronic obstructive pulmonary disease; GDS, geriatric depression scale; HNC, head and neck cancer; HR, hazard ratio; ICU, intensive care unit; MD, mean difference; MI, myocardial infarction; PHQ, patient health questionnaire; PR, prevalence ratio; PTCA, percutaneous transluminal coronary angioplasty; OR, odds ratio; RR, relative risk; SD, standard deviation; TBI, traumatic brain injury*.

### 3b Effects of Statins on the Risk of Developing Depression in Non-depressed Patients

We identified 39 records (2 meta-analyses, 23 cohort studies, 2 cohort and case-control studies, 1 cohort and cross-sectional study, 1 case-control study, 5 cross-sectional studies, and 5 case series – see Table 1) investigating the effect of statins on the risk of depression diagnosis in non-depressed participants.

### 3b-Z Meta-Analyses

One meta-analysis, by Parsaik and colleagues (73), included 4 cohort, 2 nested case-control, and 1 cross-sectional studies on a total of 9,187 non-depressed participants with median follow-up of 5 years. The pooled adjusted odds ratio (OR) of depression for statin users compared to non-users was 0.68 (95% CI = 0.52–0.89), showing statin users were 32% less likely to develop depression. One of the included studies that reported lack of association (102) accounted for most of the meta-analysis' heterogeneity (Cochran's *Q*-test *P* = 0.014, *I*^2^ = 55%). When it was removed from the analysis, the antidepressant effect of statins was stronger (OR = 0.63, 95% CI = 0.43–0.93) and heterogeneity decreased (Cochran's *Q*-test *P* = 0.40, *I*^2^ = 2%) (73). A more recent and larger meta-analysis of 13 observational studies and 5,035,070 participants reported comparable results (OR = 0.85; 95% CI = 0.72–0.99); however, no association between statin use and depression risk when the trim-and-fill analysis (i.e., a method to correct for publication bias) was used (OR = 0.87, 95% CI = 0.74–1.02), which suggests that some smaller studies with negative results may have not been published (72).

### 3b-II Cohort Studies

Of the 26 cohort studies, 14 used a prospective cohort and 12 a historical cohort design.

Nine studies investigated the association between use vs. non-use of statins in non-psychiatric populations with non-specific physical comorbidities; of these studies, half found no association between statins and depression. A small study on 1,631 adults followed for 5.2 years found no evidence of association between self-reported statins use and a formal diagnosis of depression (HR = 1.25, 95% CI = 0.73–2.14) (77). A strong protective effect (HR = 0.20, 95% CI 0.04–0.85), of statins or aspirin use on the risk of developing depression was confirmed in 345 women aged 50+ years (89). In contrast, a study by Williams and colleagues did not find any significant association (HR = 0.55, 95% CI = 0.23–1.32) between the use of either statins or aspirin and further diagnosis of mood disorder in a sample of 836 men (94). Lack of association (OR = 1.02, 95% CI = 0.94–1.11) between statins use and diagnosis of depression or bipolar disorder was also reported in a study of 13,944 adults followed for 4.5 years (85). A large study on 129,288 statin users and 600,241 matched non-users followed for 4.3 years did not identify any association (HR= 1.01, 99% CI= 0.96–1.06) between statin use and initiation of antidepressant treatment (91). A more recent study of 193,977 statin users and 193,977 matched non-users showed an increased risk of depression diagnosis (HR= 1.33, 95% CI = 1.31–1.35) in statin users, though this association became non-significant when adjusting for antidepressant use (84). Conversely, a study on prescription data from 497,080 statin users found that use of statins decreased the rate of incident depression (Trend test for statin prescription = 0.92, 95% CI = 0.92–0.95) (81), confirming the results of a previous nationwide cohort study (*N* = 4,607,990) (OR = 0.92, 95% CI = 0.89–0.96) (90). The latter article also indicated a protective effect for simvastatin (OR = 0.93, 95% CI = 0.89–0.97) and a harmful effect for atorvastatin (OR = 1.11, 95% CI = 1.01–1.22) on the risk of developing depression (despite both being lipophilic molecules) (90), whereas another study on 299,298 participants comparing lipophilic vs. hydrophilic statins highlighted an increased risk of diagnosing depression for simvastatin only (HR = 1.09, 95% CI = 1.02–1.16), but not when all lipophilic statins were compared to hydrophilic ones (HR = 1.05 95% CI = 1.00–1.10) (76). A recent within-subject epidemiological study conducted on a large nationwide register of 1,149,384 statin users followed for 8 years found that presentation for depressive disorders was less frequent during periods on statins, compared to periods off statins (HR = 0.91, 95% CI = 0.88–0.94) (87).

The remaining cohort studies focussed on groups of patients with specific physical illness. Five studies included patients with heart conditions (42, 88, 92, 95, 98). One large historical cohort study on 91,842 patients with acute coronary syndrome (ACS) and 91,860 non-ACS controls found that statins use was associated with decreased risk of both early (within 1 year) (HR = 0.94, 95% CI = 0.86–0.94) and late (within 12 years) (HR = 0.96, 95% CI = 0.82–0.90) depression, but only in the ACS patients (95). The other studies on cardiological patients were prospective and included smaller samples between 193 and 711 participants. Stafford and Berk (92) reported decreased risk of post-discharge depression at 9 months (OR = 0.21, 95% CI = 0.052–0.876). Another study found lower incidence of depression at 1 year follow-up in ACS patients taking statins (23.3%) compared to statin non-users (32.8%) (42). Two studies on patients with coronary artery disease reported a reduced risk of depressive illness as measured with mood questionnaires at 6 years (OR = 0.62, 95% CI = 0.41–0.95) (88) and 4 years (OR = 0.63, 95% CI = 0.43–0.93) (98).

Five studies were conducted on stroke patients (74, 79, 80, 82, 96). One found increased risk of post-stroke depression among statin users compared to non-users (*N* = 11,218, HR = 1.59, 95% CI = 1.30–1.95) (80). A larger study on 70,706 stroke patients found no effect (OR = 0.99, 95% CI = 0.95–1.03) of statin prescription on self-reported low mood or antidepressant use at 3 months follow-up (74). The remaining reports indicated a beneficial effect of statins on depression at 1 year follow-up: two were on small samples of 288 (OR = 0.54, 95% CI = 0.49–0.87) (82) and 286 participants (Wald = 8.477, *P* = 0.004) (79), whereas the third was conducted on a large historical cohort of 147,487 stroke patients and 160,235 matched individuals, and showed a risk reduction among both stroke patients (HR = 0.90, 95% CI = 0.87–0.93) and non-stroke patients (HR = 0.90, 95% CI = 0.86–0.94) (96). Chuang and colleagues investigated the relationship between hyperlipidaemia, statins use, and depression: 26,852 hyperlipidaemic and 107,408 non-hyperlipidaemic patients were compared, showing a lower risk of depression among hyperlipidaemic participants who received statins (HR = 0.81, 95% CI = 0.69–0.96), but in the non- hyperlipidaemic (75).

Two studies were conducted on survivors of traumatic brain injury. One found that statin use was associated with fewer depression diagnoses at 6 months (*N* = 100,515, RR = 0.85, 95% CI = 0.79–0.90) (83). The other (*N* = 3,792) reported higher risk of depression in patients that were also hyperlipidaemic vs. normolipidaemic (HR = 1.61, 95% CI = 1.03–2.53), but no significant difference between patients treated with statins or not, regardless of their hyperlipidaemic (HR = 0.63, 95% CI = 0.34–1.17) or normolipidaemic (HR = 1.02, 95% CI = 0.55–1.89) status (93).

One study conducted on 9,139 patients affected by asthma-COPD overlap syndrome and found that statin users were at decreased risk of depression for up to 11 years compared to non-users (HR = 0.36, 95% CI = 0.25–0.53) (97). Finally, two studies on 408 hyperlipidaemic head and neck cancer patients (HR = 0.85, 95% CI = 0.46–1.57) (78) and 12,176 ICU patients (RR = 1.04, 95% CI = 0.96–1.13) (86) showed no effect of statins on the risk of depression.

### 3b-III Case-Control Studies

Two cohort studies on non-depressed patients also included a case-control analysis: one reported non-significant results (*N* = 345 females, OR = 0.13, 95% CI = 0.02–1.02) (89), whilst the other showed a strong protective effect of statins (*N* = 937 males, OR = 0.1, 95% CI = 0.1–0.4) (94). Another case-control study on 366 hyperlipidaemic participants reported decreased risk of new onset depression in statin users vs. non-users (OR = 0.4, 95% CI = 0.2–0.9) (33).

### 3b-IV Cross-Sectional Studies

Six cross-sectional studies were retrieved. One showed increased absenteeism from work due to depression among employees reporting statin use (*N* = 17,244, Prevalence Ratio = 2.18, 95% CI = 1.18–4.03) (100). Another within-subjects study found no significant change (RR = 1.06, 95% CI = 0.79–1.45) in the redemption of antidepressants prescription before vs. after the initiation of statins (102). One article on patients with heart conditions found a reduction (*N* = 965, OR = 0.66, 95% CI = 0.45–0.98) (88) in risk of developing depression in statin users. The remaining studies involved elderly participants. Outcomes based on a Geriatric Depression Scale (GDS) or a Centre for Epidemiologic Studies – Depression (CES-D) score above threshold for diagnosis of depression were reported in three studies with conflicting results: one showed a decreased prevalence of depression in statin users (*N* = 2,804, OR = 0.71, 95% CI = 0.52–0.97) (101), another reported no difference in prevalence among South Asian and African-Caribbean statin users (*N* = 695, OR = 1.67, 95% CI = 0.97–2.88) nor White Europeans (*N* = 638, OR = 0.54, 95% CI = 0.26–1.13) (103) though a significant ethnicity-statin interaction (*P* = 0.041), while a further study in 19,114 elderly community-dwelling participants found no association (OR = 1.09, 95% CI = 0.98–1.20) (99).

### 3b-V Case Series

We retrieved 5 case series that we report for completeness. Tatley and colleagues investigated reports from the New Zealand Centre for Adverse Reaction Monitoring and found that, of the 203 reports of psychiatric adverse events associated with statins, 67 concerned mood disorders (108). Another record reported 12 cases of onset of violent ideation, irritability, depression, and suicide that resolved upon statins discontinuation and recurred when re-challenging (104). Two older case series included 8 reports of female patients suffering depressive and psychotic symptoms after initiation of pravastatin and simvastatin that resolved after discontinuation (105, 106); a similar clinical course was described in another case series concerning 2 males treated with lovastatin (107).

### 3c Effects of Statins on Depressive Symptoms Scores in Non-depressed Patients

We identified 22 records (3 meta-analyses, 13 clinical trials, 3 cohort studies, 3 cross-sectional studies – see Table 2) investigating the effect of statins on depressive symptoms scores in non-depressed participants.

**Table 2:** Overview of studies regarding the effects of statins on depressive symptoms scores in non-depressed participants.

**Publication**	**Study design**	**Population**	**Intervention/** **exposure**	**Comparison**	**Follow-up**	**Primary outcomes**	**Major Findings**	**Association**
**Meta-analysis**								
Köhler-Forsberg et al. (84)	Meta-analysis	7 RCTs, 1,576 depressed and non-depressed participants	Statin add-on or monotherapy	Placebo	6 weeks to 4 years	HDRS, CES-D, GHQ	SMD = −0.26 95% CI = −0.48 to −0.04 *P* = 0.02	**+**
O'Neil et al. (109)	Meta-analysis	7 RCTs, 2,105 participants	Simvastatin, lovastatin, pravastatin	Placebo	4 weeks to 4 years	HDRS, MSQ, BSI, HADS, BDI, GHQ, CES-D	SMD= −0.43 95% CI = −0.61 to −0.24 *P* = NR	**=**
Yatham et al. (110)	Meta-analysis	10 RCTs, 2,517 depressed and non-depressed participants	Atorvastatin, simvastatin, lovastatin, pravastatin	Placebo	6 weeks to 4 years	HDRS, HADS, CES-D, GHQ, POMS	SMD= −0.309 95% CI = −0.525 to −0.094 *P* = 0.005	**+**
**Randomised controlled trials**								
Carlsson et al. (111)	RCT crossover	41 hyperlipidaemic adults aged >/=70 years	Pravastatin 20 mg pravastatin 20 mg + tocopherol 400 IU	Placebo + tocopherol pravastatin + tocopherol	1 year	GDS	[From KKöhler-Forsberg (84)] SMD = 0.09 95% CI = −0.57 to 0.76 *P* = 0.622	**=**
Chan et al. (112)	RCT	140 secondary progressive multiple sclerosis patients	Simvastatin 80 mg	Placebo	2 years	HDRS	SMD = −1.0 95% CI = −3.2 to 1.2 *P* = 0.37	**=**
Gengo et al. (113)	RCT crossover	36 hyperlipidaemic patients	Lovastatin 40 mg, pravastatin 40 mg	Placebo	4 weeks	POMS	SMD= −0.633 95% CI = −1.213 to −0.053 *P* = 0.032	**+**
Harrison and Ashton (114)	RCT crossover	25 healthy volunteers	Simvastatin 40 mg, pravastatin 40 mg	Placebo	4 weeks	HADS	SMD= 0.048 95% CI = −0.507 to 0.602 *P* = 0.866	**=**
Hyyppä et al. (115)	RCT crossover	120 hyperlipidaemic men aged 35–64 years	Simvastatin 20 mg	Placebo	24 weeks	BDI	MD = 0.06 95% CI = 0.01–0.12 *P* = 0.01590	**–**
Krysiak et al. (116)	Non-randomised non-controlled trial	14 hyperlipidaemic women, 14 normolipidaemic women	Atorvastatin 20−40 mg in hyperlipidaemia	No treatment in normolipidaemia	24 weeks	BDI	Baseline: 11.6 (3.7) vs. 7.6 (3.9) SMD = NR 95% CI = NR *P* < 0.05 End of study: 9.4 (3.0) vs. 8.0 (4.3) SMD = NR 95% CI = NR *P* = NR	**+**
Morales et al. (117)	RCT	80 older adults aged >/=65 years	Simvastatin	Placebo	15 weeks	CES-D	SMD = 0.00 95% CI = −0.46 to 0.46 *P* = NR	**=**
Muldoon et al. (118)	RCT	209 hyperlipidaemic adults	Lovastatin 20 mg	Placebo	6 months	HDRS	SMD = 0.21 95% CI = −0.07 to 0.49 *P* > 0.2	**=**
Ormiston et al. (119)	Non-controlled trial	12 healthy volunteers	Atorvastatin 10–20 mg, lovastatin 20–40 mg	–	1 year	BDI	*T* = 2.27, df = 11, *P* < 0.05	**+**
Robertson et al. (120)	RCT	52 mild TBI patients aged 18–50 years	Atorvastatin 1mg/kg/die (up to 80 mg/die)	Placebo	3 months	CES-D	SMD = 0.05 95% CI = −0.495 to 0.595 *P* = 0.857	**=**
Santanello et al. (121)	RCT	431 adults aged >/= 65 years	Lovastatin 20–40 mg	Placebo	6 months	CES-D	SMD = −0.08 95% CI = −0.29 to 0.14 *P* = 0.53	**=**
Stewart et al. (122)	RCT	1,130 adults with CAD and hyperlipidaemia	Pravastatin 40 mg	Placebo	4 years	GHQ	MD = 0.49 95% CI = −0.30 to 1.28 *P* = 0.23	**=**
Wardle et al. (123)	RCT	621 adults with CAD aged 40–75 years	Simvastatin 20–40 mg	Placebo	152 weeks	POMS	SMD = −0.405 95% CI = −0.596 to −0.213 *P* = 0.000	**=**
**Cohort studies**								
Al Badarin et al. (124)	Cohort	1,691 ACS patients	Statin prescription	No prescription	1 year	PHQ-8	MD = −0.05 95% CI = −0.67 to 0.58 *P* = 0.88	**=**
Feng et al. (125)	Cohort	1,803 adults aged >/=55 years	Statin use	No use	1.5 years	GDS	Regression coefficient = −0.12 95% CI = NR *F* = 1.44 *P* = 0.23	**=**
Hoogwegt et al. (126)	Cohort	409 ICD-implanted patients	Statin use	No use	1 year	HADS	MD = −0.97 95% CI = −1.99 to 0.05 *P* = 0.6	**=**
**Cross sectional studies**								
Agostini et al. (127)	Cross sectional	756 community-dwelling veterans aged >/= 65 years	Statin use	No use	–	CES-D	SMD = −0.18 95% CI = −0.69 to 0.33 *P* = 0.49	**=**
Mandas et al. (128)	Cross sectional	329 adults with dyslipidaemia aged >/= 65 years	Statin use	No use	–	GDS	SMD = 0.4573 95% CI = NR *P* = 0.01828	**–**
Olson et al. (129)	Cross sectional	525 women undergoing coronary angiography	Cholesterol-lowering drug use (self-reported)	No use	–	BDI	BDI: SMD = NR 95% CI = NR *P* = 0.94	**=**

*The effect size of the main findings, either extracted from the study or calculated by the authors. Where this was not possible, we report the raw data.

*ACS, acute coronary syndrome; BDI, beck depression inventory; BSI, brief symptom inventory; CAD, coronary artery disease; CES-D, centre for epidemiological studies – depression scale; CI, confidence interval; GDS, geriatric depression scale; GHQ, general health questionnaire; HADS, hospital anxiety and depression scale; HDRS, hamilton depression rating scale; ICD, implantable cardioverter-defibrillator; MD, mean difference; MSQ, mood states questionnaire; PHQ, patient health questionnaire; POMS, profile of mood scores; PR, prevalence ratio; OR, odds ratio; RCT, randomised controlled trial; SD, standard deviation; SMD, standardised mean difference; TBI, traumatic brain injury*.

### 3c-I Meta-Analyses

Of the three meta-analyses we identified, two reported the antidepressant effect of statins and one found no association. A meta-analysis of 7 RCTs (*N* = 2105) with follow-up between 4 weeks and 4 years reported no overall difference in depressive scores between participants receiving statins or placebo (SMD = −0.08, 95% CI = −0.29 to 0.12) (109). A more recent meta-analysis assessed the effects of all anti-inflammatory drugs in a mixed sample of non-depressed patients with baseline depressive symptoms and patients with a diagnosis of depression; with regards to the statins' trials (either in add-on to antidepressants, or in monotherapy), 7 RCTs (*N* = 1,576) were retrieved by the author and indicated an antidepressant effect of statins (SMD = −0.26, 95% CI = −0.48 to −0.04) (138). Another meta-analysis on statins only, again on a mixed sample of non-depressed and depressed patients, included 10 RCTs (*N* = 2,517) and confirmed that statins reduced depressive scores (SMD = −0.309, 95% CI = −0.525 to −0.094), though with high heterogeneity and poorly determined risk of bias (110). Interestingly, despite the essentially overlapping inclusion/exclusion criteria of these meta-analyses, only one study was included in all three of them (122). Two studies (115, 117) were only included in the older one (109). Two studies were only included by Köhler-Forsberg and colleagues (111, 121), and one only by Yatham and colleagues (120). Four studies were included by Yatham and colleagues and O'Neil and colleagues, but not by Köhler-Forsberg and colleagues (113, 114, 118, 123).

### 3c-II Clinical Trials

We identified 11 RCTs evaluating the effect of statins on mood scores in non-depressed participants. Two RCTs demonstrated opposing significant effects of statin use on depressive scores. A crossover RCT including 36 hypercholesteraemic patients followed-up for 4 weeks showed a reduction in depressive scores associated with statins' use (SMD = −0.633, 95% CI = −1.213 to −0.053) (113), whereas another crossover RCT assessing the separate and combined effects of simvastatin and Mediterranean-type diet in 120 hyperlipidaemic men followed-up for 24 weeks highlighted an increase in depressive scores in the statin group [Mean Difference (MD) = 0.06, 95% CI = 0.01–0.12] (115).

None of the remaining 9 RCTs reported significant changes in depressive scores associated with statins. One study on 25 healthy volunteers found no effect of simvastatin or pravastatin compared to placebo on depressive symptoms at 4 weeks (MD = 0.05, 95% CI = −0.51 to 0.60) (114). Similar results were reported in a sample of 209 hyperlipidaemic adults treated with lovastatin vs. placebo for 6 months (SMD = 0.21, 95% CI = −0.07 to 0.49) (118). Two further RCTs were conducted on larger samples of individuals with heart conditions (*N* = 1,130 and *N* = 621) and had longer follow-up (4 years and 152 weeks). Both Stewart and colleagues (122) and Wardle and colleagues (123), respectively, showed no differences in depressive scores depending on intervention (MD = 0.49, 95% CI = −0.30 to 1.28; χ2 heterogeneity = 1.66, linear trend = 0.08). Three trials on 431 (121), 80 (117), and 41 (111) older adults, respectively, found no differences (SMD = −0.08, 95% CI = −0.29 to 0.14; SMD = 0.00, 95% CI = −0.46 to 0.46; SMD = 0.09, 95% CI = −0.57 to 0.76) at 15 weeks, 6 months, and 1 year in depressive scores. Chan and colleagues conducted a trial on 140 patients with multiple sclerosis and found no significative difference in depressive scores (SMD = −1.0, 95% CI = −3.2 to 1.2) between simvastatin treatment and placebo at 2 years (112). Similar results (SMD = 0.05, 95% CI = −0.495 to 0.595) were reported in 52 mild traumatic brain injury patients using atorvastatin for 3 months (120).

Two further trials were non-controlled and/or non-randomised. One small (*n* = 12) double blind pilot study did not include a placebo arm, and reported an improvement in depressive scores (*t* = 2.27, df = 11) following treatment with statins (119). Another small non-randomised non-placebo controlled study on 14 hypercholesteraemic women receiving 24 weeks treatment with atorvastatin and 14 normolipidaemic patients left untreated found that the depressive scores at baseline were significantly higher among the hypercholesteraemic (*p* < 0.05) and treatment with statins normalised depressive scores (116).

### 3c-III Cohort Studies

All 3 cohort studies identified showed no effect of statins on depressive scores. A cohort study on 1,691 patients with acute myocardial infarction showed that statin prescription did not affect the natural decrease in depressive scores (MD = −0.05, 95% CI = −0.67 to 0.58) at 1 year follow-up (124). Similar results (MD = −0.97, 95% CI = −1.99 to 0.05) were reported by Hoogwegt and colleagues in 409 ICD-implanted patients (126). Another study on 1,803 elderly participants concluded that statin use did not correlate with increased scores of the Geriatric Depression Scale (*p* = 0.23) at 1.5 years follow-up, though *post hoc* analyses suggested a protective effect of statins in female participants and an opposite effect in men (125).

### 3c-IV Cross-Sectional Studies

Of the three cross-sectional studies, two did not show any association between statins use and depressive scores: one was conducted on 756 elderly veterans (beta = −0.18, 95% CI = −0.69 to 0.33) (127) and the other on 525 female patients with heart conditions (*P* = 0.94) (129). The remaining study was again in elderly dyslipidaemic patients but this one reported an increase in depressive scores in statin-users (*N* = 329, *P* = 0.018) (128).

### 3d Effects of Statins on the Risk of Developing Depressive Episodes or on Depressive Symptoms Scores in Depressed Patients

We identified 11 records (3 meta-analysis, 6 clinical trials, 2 cohort studies – see Table 3) investigating the effect of statins on depression in depressed patients.

**Table 3:** Overview of studies regarding the effects of statins on the risk of developing depressive episodes or on depressive symptoms scores in depressed patients.

**References**	**Study design**	**Population**	**Intervention/** **exposure**	**Comparison**	**Follow-up**	**Primary outcomes**	**Major Findings**	**Association**
**Meta-analysis**								
Bai et al. (19)	Meta-analysis	3 RCTs, 166 MDD patients	TAU + Statin	TAU + placebo	6–12 weeks	HDRS	SMD = −0.65 95% CI = −0.96 to −0.33 *P* < 0.0001	**+**
De Giorgi et al. (71)	Meta-analysis	4 RCTs, 255 MDD patients	TAU + Statin	TAU + placebo	8 weeks	HDRS/MADRS	SMD = −0.48 95% CI = −0.74 to −0. 22 *P* = NR	**+**
Salagre et al. (130)	Meta-analysis	3 RCTs, 165 MDD patients	TAU + Statin	TAU + placebo	6–12 weeks	HDRS	SMD = −0.73 95% CI = −1.04 to −0.42 *P* < 0.001	**+**
**Randomised controlled trials**								
Abbasi et al. (131)	RCT	46 post CABG patients with mild to moderate depression	Simvastatin 20 mg	Atorvastatin 20 mg	6 weeks	HDRS	SMD = 3.63 95% CI = 0.44–6.51 *P* = 0.03	**+**
Berk et al. (132)	RCT	130 MDD patients aged 15–25 years	TAU + rosuvastatin 10 mg	TAU + placebo	12 weeks	MADRS	SMD = −4.2 95% CI = −9.1 to 0.6 *P* = 0.089	**=**
Ghanizadeh and Hedayati (133)	RCT	68 MDD patients	Fluoxetine 40 mg + lovastatin 30 mg	Fluoxetine 40 mg + placebo	6 weeks	HDRS	SMD = −0.77 95% CI = −1.30 to −0.24 *P* < 0.001	**+**
Gougol et al. (134)	RCT	48 MDD patients	Fluoxetine 20 mg + simvastatin 20 mg	Fluoxetine 20 mg + placebo	6 weeks	HDRS	SMD = −0.73 95% CI = −1.34 to −0.11 *P* = NR	**+**
Haghighi et al. (135)	RCT	60 MDD patients	Citalopram 40 mg + atorvastatin 20 mg	Citalopram 40 mg + placebo	12 weeks	HDRS	SMD= −0.70 95% CI = −1.22 to −0.18 *P* = NR	**+**
Soh et al. (136)	RCT	60 patients with mood disorder	Lithium + atorvastatin 20 mg	Lithium + placebo	12 weeks	Relapse (MADRS≥10)	χ2 (1) = 0.148 95% CI = NR *P* = 0.70	**=**
**Cohort studies**								
Kim et al. (68)	Cohort	300 patients with comorbid ACS and depression	Escitalopram + statin, statin-only use	Escitalopram-only, placebo-only	1 year	Response (HDRS, BDI)	HDRS response: OR = 2.23 95% CI = 1.11–4.51 *P* = 0.025 BDI response: OR = 2.82 95% CI = 1.35–5.90 *P* = 0.006	**+**
		146 patients with comorbid ACS and depression	Statin use	TAU	1 year	Response (HDRS, BDI)	HDRS response: OR = 1.19 95% CI = 0.45–3.18 *P* = 0.726 BDI response: OR = 0.89 95% CI = 0.36–2.22 *P* = 0.798	**=**
Köhler et al. (137)	Historical cohort	872,216 SSRI users	SSRI + statin use	SSRI-only use	3 years	Depressive episode (hospital contact)	HR = 0.64 95% CI = 0.55–0.75 *P* = NR	**+**

*The effect size of the main findings, either extracted from the study or calculated by the authors. Where this was not possible, we report the raw data.

*ACS, acute coronary syndrome; BDI, beck depression inventory; CABG, coronary artery bypass graft; CI, confidence interval; HDRS, hamilton depression rating scale; HR, hazard ratio; MADRS, montgomery-åsberg depression rating scale; MD, mean difference; MDD, major depressive disorder; PR, prevalence ratio; OR, odds ratio; RCT, randomised controlled trial; SD, standard deviation; SMD, standardised mean difference; SSRI, selective serotonin reuptake inhibitors; TAU, treatment as usual*.

### 3d-I Meta-Analyses

Two meta-analyses reported results on the effect of statins as add-on treatment in depressed patients, with similar results. They both included 3 RCTs (133–135) and found a significant improvement in depressive symptoms [SMD = −0.73, 95% CI = −1.04 to −0.42 (130); SMD = −0.65, 95% CI = −0.96 to −0.33 (19)] associated with statins add-on. A more recent meta-analysis added a further RCT (132) and confirmed the above results (SMD = −0.48, 95% CI = −0.74 to −0.22], whilst also supporting the acceptability, tolerability, and safety of statins in the treatment of depression (71).

### 3d-II Clinical Trials

We found 6 clinical trials investigating the effect of statins in depressed patients (3 of which were included in the meta-analyses commented on above). In the oldest of these RCTs, 68 depressed patients were randomised to 6 weeks of either fluoxetine plus lovastatin or fluoxetine plus placebo: depressive scores decreased significantly in both groups, but more noticeably in the treatment group [mean change = 12.8 (SD = 6.3) vs. 8.2 (SD = 4.0, *t* = 3.4, df = 60)] (133). A similar 6 week trial on simvastatin randomised 48 depressed patients and found comparable results (SMD = 4.81, *P* = 0.02), though remission rates were not significantly different (59 vs. 45%, *P* = 0.36) (134). In a 12-week trial, 60 depressed patients received citalopram for 1 week and were then randomised to either atorvastatin or placebo adjunction. Results showed significantly lower depressive scores [Time × Group interaction: *F*_(3,174)_ = 8.93] and increased partial remission (OR = 8.83, 95% CI = 1.02–76.96) for the statin group (135). A recent RCT included 130 MDD patients aged 15 to 25 years old, who were randomised to receive either treatment as usual (TAU) plus placebo, or TAU plus aspirin, or TAU plus rosuvastatin. Differences in changes in Montgomery-Asberg Depression Rating Scale (MADRS) scores were not significant when comparing rosuvastatin and placebo group (SMD = −4.2, 95% CI = −9.1 to 0.6, *P* = 0.089) nor when comparing rosuvastatin vs. aspirin groups (SMD = −6.4, 95% CI = −11.7 to 1.2) (132). The most recent RCT compared the relapse risk in a sample of 60 BD or MDD patients randomised to either lithium plus atorvastatin or lithium plus placebo, and reported non-significant effect (χ2_(1)_ = 0.148, *P* = 0.70) (136).

We also retrieved a RCT comparing the antidepressant effects of simvastatin vs. atorvastatin, without a placebo control, in 46 post-CABG patients with comorbid mild to moderate depression. Depressive scores at 6 weeks decreased more prominently in the simvastatin group (SMD = 3.63, 95% CI = 0.44–6.51, *P* = 0.03) (131).

### 3d-III Cohort Studies

Two cohort studies assessed the effect of statins in depressed patients. Kim and colleagues analysed 1-year follow-up data of a previous 24-week RCT of escitalopram in 300 patients with comorbid ACS and depression. Incidental statin-users showed higher response rates on both the Hamilton Depression Rating Scale (HAM-D) (OR = 2.23, 95% CI = 1.11–4.51) and the Beck's Depressive Inventory (BDI) (OR = 2.82, 95% CI = 1.35–5.90) at 1 year (68). The same paper however found no differences in response (HAM-D: OR = 1.19, 95% CI = 0.45–3.18; BDI: OR = 0.89, 95% CI = 0.36–2.22) when considering the effect of statins' monotherapy in patients who were not on antidepressants (*N* = 146). A higher response rate, though only on the HAM-D, was observed in users of lipophilic statin vs. all others statins users (OR = 2.91, 95% CI = 1.21–6.99) (68). Finally, a large (*N* = 872,216) historical cohort study compared several outcomes associated with depression in a group of SSRIs-plus-statins users against a group of SSRIs-only users and found a significantly lower risk for psychiatric hospital contacts (HR = 0.75, 95% CI = 0.69–0.82) and psychiatric hospital contacts specifically due to depression (HR = 0.64, 95% CI = 0.55–0.75) among statin users. No significant differences were reported in all-cause mortality (HR = 1.04, 95% CI = 0.96–1.12) and suicidality (HR = 0.85, 95% CI = 0.61–1.18) (137).

### 3e Effects of Statins on Depressive-Inflammatory Symptoms in Depressed Patients

Fifteen of the studies described above also investigated the effect of statins on specific depressive-inflammatory symptoms in patients with depression (see Table 4).

**Table 4:** Overview of studies regarding the effects of statins on depressive-inflammatory symptoms.

**Publication**	**Study design**	**Population**	**Intervention/** **exposure**	**Comparison**	**Follow-up**	**Primary outcomes**	**Major Findings**	**Association**
**ANHEDONIA**								
**Psychomotor retardation**								
**Randomised Controlled Trials**								
Harrison and Ashton (114)	RCT crossover	25 healthy volunteers	Simvastatin 40 mg, pravastatin 40 mg	Placebo	4 weeks	DSST	Pravastatin: mean = 74.3 95% CI = 70.3–78.3 Simvastatin: mean = 74.6 95% CI = 70.3–78.9 Placebo: mean = 74.6 95% CI = 70.9–78.3 SMD = NR 95% CI = NR *P* = NR	**=**
Muldoon et al. (118)	RCT	209 hyperlipidaemic adults	Lovastatin 20 mg	Placebo	6 months	DSST	MD = 0.06 95% CI = 0.01–0.12 *P* = 0.016	**+**
Santanello et al. (121)	RCT	431 adults aged >/= 65 years or older	Lovastatin 20, lovastatin 40 mg	Placebo	6 months	DSST	Mean (SD) = Placebo 0.33 (13.6) Lovastatin 20 mg −0.80 (13.28) Lovastatin 40 mg 1.66 (8.98) SMD = NR 95% CI = NR *P* = 0.66	**=**
**ANXIETY**								
**Randomised controlled trials**								
Berk et al. (132)	RCT	130 MDD patients aged 15–25 years	TAU + rosuvastatin 10 mg	TAU + placebo	12 weeks	GAD	SMD = −0.6 95% CI = −1.7 to 3.0 *P* = 0.684	**=**
Harrison and Ashton (114)	RCT crossover	25 healthy volunteers	Simvastatin 40 mg, pravastatin 40 mg	Placebo	4 weeks	HADS (anxiety subscale)	Pravastatin: mean score = 3.2 95% CI = 2.0–4.4 Simvastatin: mean score = 2.5 95% CI = 1.7–3.3 Placebo mean score = 3.1 95% CI = 2.2–4.0 SMD = NR 95% CI = NR *P* = NR	**=**
Hyyppä et al. (115)	RCT crossover	120 hyperlipidaemic men aged 35–64 years	Simvastatin 20 mg	Placebo	24 weeks	BDI (anxiety items)	Mean change= 0.00 95%CI = −0.05 to 0.05 P= 0.9474	**=**
Stewart et al. (122)	RCT	1,130 adults with CAD and hyperlipidaemia	Pravastatin 40 mg	Placebo	4 years	GHQ (anxiety items)	MD= NR 95% CI = NR P= “non-significant”	**=**
Wardle et al. (123)	RCT	621 adults with CAD aged 40–75 years	Simvastatin 20–40 mg	Placebo	152 weeks	POMS (tension/anxiety items)	X^2^ = 3.57 95% CI = NR *P* = “non-significant”	**=**
**Cohort studies**								
Hoogwegt et al. (126)	Cohort	409 ICD-implanted patients	Statin use	No use	1 year	HADS (anxiety subscale)	MD = −0.81 95% CI = −1.80 – 0.18 *P* = 0.11	**=**
Molero et al. (87)	Historical cohort	1,149,384 statin users aged >/= 15 years	Statin use	No use	8 years	Diagnosis of anxiety disorder (unplanned hospital visit or specialised outpatient care)	HR = 0.99 95% CI = 0.95–1.02 *P* = NR	**=**
Young-xu et al. (98)	Cohort	371 CAD patients	Statin use	No use	4 years	Diagnosis of anxiety (Kellner Symptom questionnaire >/=8)	OR = 0.69 95% CI = 0.47 – 0.99 *P* = NR	**+**
**SLEEP**								
**Randomised controlled trials**								
Carlsson et al. (111)	RCT crossover	41 hyperlipidaemic adults aged >/= 70 years	Pravastatin 20 mg pravastatin 20 mg + tocopherol 400 IU	Placebo + tocopherol pravastatin + tocopherol	1 year	SDS	SMD = NR 95% CI = NR *P* = 0.761	**=**
Harrison and Ashton (114)	RCT crossover	25 healthy volunteers	Simvastatin 40 mg, pravastatin 40 mg	Placebo	4 weeks	LSQ	Pravastatin: mean = 51.4 95% CI = 48.4–54.6 Simvastatin: mean = 47.0 95% CI = 44.9–49.1 Placebo: mean = 50.1 95% CI = 46.4–53.8 SMD = NR 95% CI = NR *P* = NR	**=**
Santanello et al. (121)	RCT	431 adults aged >/= 65 years	Lovastatin 20–40 mg	Placebo	6 months	SDS	Mean change (SD) = Placebo −0.07 (2.39) Lovastatin 20 mg −0.05 (2.53) Lovastatin 40 mg 0.46 (3.07) SMD = NR 95% CI = NR *P* = 0.93	**=**
Wardle et al. (123)	RCT	621 adults with CAD aged 40–75 years	Simvastatin 20–40 mg	Placebo	152 weeks	Sleep symptoms report	Prevalence difference= −5.3% (statin users) MD = NR 95% CI = NR *P* = NR	**+**

*The effect size of the main findings, either extracted from the study or calculated by the authors. Where this was not possible, we report the raw data.

*BDI, beck depression inventory; CAD, coronary artery disease; CI, confidence interval; DSST, digit symbol substitution test; GHQ, general health questionnaire; HADS, hospital anxiety and depression scale; HR, hazard ratio; ICD, implantable cardioverter-defibrillator; LSQ, leeds sleep questionnaire; MD, mean difference; MDD, major depressive disorder; POMS, profile of mood scores; PR, prevalence ratio; OR, odds ratio; RCT, randomised controlled trial; SD, standard deviation; SDS, sleep dysfunction scale; SMD, standardised mean difference; TAU, treatment as usual*.

### 3e-I Anhedonia

We could not identify any paper specifically addressing anhedonia.

### 3e-II Psychomotor Retardation

Three of the mentioned RCTs investigated the effects of statins on measures of psychomotor retardation. A study conducted on 209 hyperlipidaemic adults reported a statistically better psychomotor speed for the placebo group vs. lovastatin-treated subjects (Z score = 0.17, 95% CI = 0.05–0.28) (118). The two remaining studies reported no significant difference in Digit Symbol Substitution Test (DSST) scores between statin and placebo groups (114, 121).

### 3e-III Anxiety

Eight studies measured anxiety as well as depressive scores. The most recent RCT found no significant difference in Generalised Anxiety Disorder 7-items scale score reduction between a rosuvastatin and placebo group (MD = −0.6, 95% CI = −1.7 to 3.0, *P* = 0.684) (132). A crossover RCT on 25 healthy volunteers did not show any difference on the anxiety items of a hospital anxiety and depression scale between pravastatin (mean = 3.2, 95% CI = 2.0–4.4), simvastatin (mean = 2.5, 95% CI = 1.7–3.3), and placebo (mean = 3.1, 95% CI = 2.2–4.0) groups (114). Another RCT on 621 adults with increased risk of coronary artery disease (CAD) found no difference in anxiety scores (χ2 = 3.57, linear trend = 0.07) between simvastatin and placebo after a 152-week follow-up (123). Two RCTs were conducted on patients with previous ACS (*N* = 1,130) (122) and hyperlipidaemia (*N* = 120) (115), and could not find any significant effect of statins on anxiety scores (MD = 0.49, 95% CI = −0.30–1.28; MD = 0.00, 95% CI = −0.05 to 0.05, respectively). Conversely, a cohort study on 371 CAD patients showed an improvement in anxiety among statin users (OR = 0.69, 95% CI = 0.47–0.99) (98). Another cohort study conducted on patients with ICD (*N* = 409) reported non-significant differences in anxiety scores when statins were used (MD = −0.81, 95% CI = −1.80 to 0.18) (126). Last, a recent cohort study on Swedish nationwide register of statin users (*N* = 1,149,384) reported no difference in the risk anxiety disorders presentation when on statins compared to periods off statins (HR = 0.99, 95% CI = 0.95–1.02) (87).

### 3e-IV Sleep

Four papers also investigated the effect of statins on sleep symptoms in depression and found no association. Harrison and colleagues did not find significant differences on Leeds Sleep Questionnaire (LSQ) scores between simvastatin (mean = 47.0, 95% CI = 44.9–49.1), pravastatin (mean = 51.4, 95% CI = 48.4–54.6), or placebo groups (mean = 50.1, 95% CI = 46.4–53.8) (114). A positive effect of simvastatin, though of unclear statistical value, was seen in a sample of patients at increased CAD risk, with sleep disturbances reported by 48.8% of patients taking simvastatin and 54% of patients taking placebo (123). Two further RCTs on, respectively, 431 (121) and 41 (111) older adults randomised to statins or placebo did not identify any changes on a sleep dysfunction scale (*P* = 0.93; *P* = 0.76, respectively).

## Discussion

In this paper, we illustrated the mechanisms whereby statins may play a role in ameliorating the pathophysiological changes associated with depression and reported on 72 clinical studies on the effects of statins in both non-depressed and depressed patients. To our knowledge, this is the largest review to date that retrieved and discussed this extensive literature following a systematic, evidence-based methodology. Although our aim was chiefly to provide the reader with a comprehensive, descriptive overview of the available literature, the collected data allow to draw some important conclusions.

The high number of articles retrieved as well as the presence of a definite trend for larger and more robust studies emphasise the interest of the scientific community to this research area, which may have significant implications for routine clinical practise. Such awareness likely stems from two important observations: firstly, statins are among the most commonly prescribed medications (139), hence the discovery that their use is associated with either antidepressant or depressogenic effects would have a very substantial impact on public health; and secondly, the relative lack of any breakthrough development of new antidepressant drugs capable of targeting alternative biological pathways (140), or that are free from concerns about adverse events and misuse [e.g., ketamine (141)], immediately make the potential use of statins especially appealing.

The issue of whether statins are to be considered a public health concern because of a depressogenic potential in non-depressed people appears less likely, from a purely numerical perspective, by looking at the large majority of studies reporting either no effect (=29) or at best a positive effect (=25) on depression as compared to studies reporting a negative effect (=10). From this perspective, data from large epidemiological studies can be particularly informative: the latest meta-analysis of observational studies included over 5 million non-depressed participants and did not observe any negative effect of statins on the risk of receiving a diagnosis of depression (72). This result appears in contrast with a previous smaller meta-analysis that had highlighted a risk reduction for depression in statins' users (73), with such difference mainly driven by the addition of a large Danish cohort study showing an increased risk of developing depression associated with the prescription of statins (84). However, the latter authors reported that this negative effect of statins became non-significant after adjusting for clinical-demographic variables and seemed mainly driven by confounders (84); moreover, the meta-analysis by Lee and colleagues (72) did not include the more recently published results on more than 1 million statins' users showing instead a significant reduction in clinical presentations for depressive episodes when participants were taking statins compared to when they were not (87). Data on the effects of statins on depressive scores in non-depressed or mixed populations, which could be more sensitive to smaller changes as compared to new diagnosis of or presentations for depression, are only available on smaller samples mainly from RCTs (109, 110, 138) rather than larger observational studies. This is likely due to national registers not routinely recording measures on scales of depression — surely an important avenue for further research. Still, taken together, these results provide reassurance that statins are unlikely to provoke the onset of depressive syndrome in otherwise healthy populations.

When considering studies specifically directed at groups of depressed patients, the numbers are even more favourable (positive effect =8 studies, no effect =2 studies, negative effect = no studies). Perhaps unsurprisingly, this crude dichotomy suggests that statins might indeed have an antidepressant action in people suffering from depression, but they are unable to improve mood in non-depressed subjects; similar to traditional antidepressants (142). In line with this, the large meta-analysis of RCTs on a mixed population by Yatham and colleagues (110) revealed via a subgroup analysis that only the depressed sample showed significantly improved mood on statins, whereas such effect was not apparent in the non-depressed subgroup. Translating this concept into neurobiological terms, statins would be able to express an antidepressant activity only in those people who present an underlying condition, such as increased inflammation, which directly contributes to their depressive symptomatology, whereas they would have no effect or even a negative effect if they act to perturb physiological processes, such as the regulatory functions of inflammatory cytokines, that are necessary for neuronal integrity (143). To paraphrase, both “too much” and “too little” inflammation are problematic when it comes to mood homeostasis, so an anti-inflammatory drug can be beneficial only if it hits the “sweet spot” in between these two conditions (34).

It follows that, if the antidepressant effect of statins was mainly explained by their anti-inflammatory properties, only a subset of patients whose depression was related to increased inflammation (12) would benefit from their use. For example, a previous study in depressed participants showed that, whilst patients who had raised inflammatory markers appeared to improve when on the anti-cytokine medication infliximab, those who did not have features of increased baseline inflammation indeed displayed a worsening of their depressive symptoms (144). Matters are complicated further when considering that there is no real consensus about the mechanistic processes contributing to statins' activity on mood, which likely involves complex interactions between several biological systems (24); therefore, for instance, it may be important to also consider lipid profiles when exploring the effect of stains on depressive symptoms. It is therefore conceivable that the use of statins could indeed lead to insubstantial or even harmful effects on mood because of the confounding heterogeneity of non-depressed, non-inflamed, or non-dyslipidaemic participants. In this respect, it will be intriguing to learn the results of two concurrent clinical trials investigating the antidepressant potential of simvastatin in patients with depression and comorbid obesity [i.e., a condition associated with abnormal lipid metabolism, inflammation, and antidepressant treatment resistance (145)] (146), and in patients with treatment-resistant depression whilst accounting for the mediating effects of blood lipids and CRP (147).

Other demographic and clinical variables that have been linked with baseline inflammatory status could likewise play a significant role. For example, elderly people are more likely to present with higher levels of inflammation (148), and indeed the older the patient, the higher the apparent benefit of statins on depression (90), and perhaps vice versa in younger populations (132). Similarly, sex differences in immune functions might explain why women, who are generally more liable to increased activity of the immune system (149), responded more than men to the antidepressant effect of statins (125). Also, a large cohort of non-depressed participants on statin treatment following an acute coronary event, a condition associated with increased systemic inflammation, showed a reduced likelihood of developing depression, but such effect was not seen in the group without underlying coronary syndrome (95). Results in patients with post-stroke depression were more heterogeneous (74, 80, 82, 96), but when the mediating effect of the pro-inflammatory cytokine IL-6 was taken into account, an antidepressant effect of statins once again emerged (79).

Despite significant evidence connecting inflammation with the beneficial effects of statins in depression, we could not identify any study explicitly addressing whether statins affect the cardinal depressive-inflammatory symptom cluster of anhedonia (150). Although the design of a study targeting such a specific symptom may seem impractical or restrictive, its importance has been highlighted by a recent trial of the anti-inflammatory sirukumab, which failed to show any effect on total depressive scores but was associated with a significant improvement in a rating scale measuring aspects of anhedonia (151). Anhedonia is reportedly one of the most impairing depressive symptoms and responds relatively poorly to treatment with conventional antidepressants (152); therefore a beneficial activity of statins on this symptom cluster would have an important clinical impact. Evidence about the effect of statins on psychomotor retardation, a symptom closely related to anhedonia (153) probably due to a shared common pathway involving dopaminergic dysfunction, was likewise sparse and inconsistent. Instead, more data were available for the depressive-inflammatory symptoms of anxiety and sleep disturbances. With regards to the former, despite animal studies suggesting that statins could lead to increased anxiety in rats (61), several clinical studies have shown no negative effects (87, 132), or indeed an improvement (98) of anxiety scores in statin-users. Then, contrary to a wide literature describing a potential association between statins' use and sleep problems in non-depressed populations (154), we could not identify any study that reported similar outcomes in the context of depressive symptoms, including in subgroups of elderly patients who are generally more likely to suffer from sleep difficulties (121).

Another factor potentially contributing to the heterogeneity of the published findings might be related to the notion that all statins are equally capable of expressing an antidepressant effect or indeed any neurobiological effect at all. Although most statins share similar pharmacodynamic properties, their pharmacokinetics and especially their lipophilicity (and thus arguably their potential to penetrate the blood-brain barrier) vary dramatically (155). For example, all the retrieved RCTs (133–135) that employed a lipophilic statin (respectively, lovastatin, atorvastatin, and simvastatin) in depressed patients observed an improvement of depressive symptoms, whereas the only trial that could not replicate this effect used the hydrophilic molecule, rosuvastatin (132). Interestingly, simvastatin, the most lipophilic statin, showed a more pronounced antidepressant effect compared to the less lipophilic atorvastatin in another RCT (131) and fared better than any other statin in a recent exploratory network meta-analysis (71). Likewise, evidence from most observational studies in non-depressed populations reported that simvastatin had the most beneficial effects (87, 90), though another study reported a conflicting finding (76). Again, data from the previously mentioned ongoing trials (146, 147), which are both using the highly lipophilic simvastatin, possibly corroborated by further observational studies comparing the effects of individual lipophilic and hydrophilic statins, could provide further useful evidence.

Our study has several limitations. Firstly, although we collated the largest number of studies on the effects of statins in depression to date, we did not perform any quantitative pooled analysis. Here we defer to the numerous meta-analyses published thus far (19, 72, 73, 109, 110, 130, 138); however, our aim was not to replicate the findings from such an extensive amount of previous secondary research. but rather to use a sensitive and systematic search strategy to ensure a very high level of inclusiveness. In this way we hoped to provide readers with a comprehensive, unbiased narrative review on the topic of statins and depression, complemented by the involvement of several authors in the process of literature search and data extraction. Nevertheless, it is possible that some significant records have been missed. Finally, many of the included studies, especially the older literature, did not report or were indeed lacking methodological detail, which may have affected our attempted interpretation and contextualisation of the findings. However, the identification of such methodological issues is likewise important as it will inform further research studies.

In summary, our broad evidence-based overview of the mechanisms and clinical studies on statins and their effects in depression adds to the wide literature investigating this important research and clinical subject. In view of the substantial amount of evidence suggesting an effect of statins on depressive symptoms, and the potential implications for clinical practise and public health should these effects be confirmed, we advocate for further mechanistic, observational, and interventional studies to definitively shed light on this matter.

## Data Availability Statement

The original contributions presented in the study are included in the article/Supplementary Material, further inquiries can be directed to the corresponding authors.

## Author Contributions

RD, NR, PC, and CH contributed to conception of the study. RD and NR designed the study. RD, NR, AQ, and FD contributed to the organisation of the database. RD and FD performed the literature search. NR and AQ extracted the data. RD wrote the first draught of the paper. NR devised the figures and tables. PC and CH supervised the study. All authors contributed to the article and approved the submitted version.

## Conflict of Interest

CH has received consultancy fees from P1vital, Janssen, Sage Pharmaceuticals, Zogenix, Pfizer, and Lundbeck outside of the current work. The remaining authors declare that the research was conducted in the absence of any commercial or financial relationships that could be construed as a potential conflict of interest.

## Publisher's Note

All claims expressed in this article are solely those of the authors and do not necessarily represent those of their affiliated organizations, or those of the publisher, the editors and the reviewers. Any product that may be evaluated in this article, or claim that may be made by its manufacturer, is not guaranteed or endorsed by the publisher.
