# Supplementary material for: Statins in Depression: An Evidence-Based Overview of Mechanisms and Clinical Studies
Source: Front Psychiatry. 2021 Jul 27;12:702617. doi: 10.3389/fpsyt.2021.702617 (PMC8353114; doi:10.3389/fpsyt.2021.702617)
Supplement: Supplementary file 1 [file Data_Sheet_1.DOCX]

**Supplementary materials**

1. Search strategy

(("Hydroxymethylglutaryl-CoA Reductase Inhibitors"[Mesh]) OR *statin OR statins) AND ("Depression"[Mesh] OR "Depressive Disorder"[Mesh] OR "Depressive Disorder, Treatment-Resistant"[Mesh] OR "Depressive Disorder, Major"[Mesh] OR "Sleep"[Mesh] OR "Sleep Wake Disorders"[Mesh] OR "Sleep Initiation and Maintenance Disorders"[Mesh] OR "Sleep Stages"[Mesh] OR "Sleep, REM"[Mesh] OR "Sleep Disorders, Circadian Rhythm"[Mesh] OR "Anhedonia"[Mesh] OR "Anxiety"[Mesh] OR "Anxiety Disorders"[Mesh] OR "Psychomotor Disorders"[Mesh] OR depression OR depressive OR sleep OR insomnia OR sleep disorder OR anhedonia OR anxiety OR psychomotor retardation OR psychomotor impairment OR anx* OR antidepress*))
